# Clinicopathological Factors Affecting Lymph Node Yield and Positivity in Left-Sided Colon and Rectal Cancers

**DOI:** 10.7759/cureus.19115

**Published:** 2021-10-29

**Authors:** Nikhil Nanjappa Ballanamada Appaiah, Muhammad Rafaih Iqbal, Omotara Kafayat Lesi, Sushmitha Medappa Maruvanda, Wenyi Cai, Andrien Rajakumar, Laeeq Khan

**Affiliations:** 1 General and Colorectal Surgery, Basildon and Thurrock University Hospital, Basildon, GBR; 2 Paediatrics, M. S. Ramaiah Medical College, Bangalore, IND

**Keywords:** colorectal cancer, anterior resection, crm involvement, perineural invasion, vascular invasion, open colorectal surgery, laparoscopic colorectal surgery, lymph node metastasis, lymph node status, lymph node yield

## Abstract

Background

Colorectal cancer (CRC) is a significant cause of cancer‐related deaths worldwide and is the third most common cause of cancer deaths in the UK. The status of lymph node metastasis is a key factor for predicting the prognosis of a patient's CRC.

Aims

This study aimed to analyze the demographics of left-sided colonic and rectal cancers at a single institution. We looked closely at the correlation between patient age and various histological factors. We tried to find any significant difference in lymph node yield (LNY) between laparoscopic surgery (LS) and open surgery (OS). We aimed to identify any statistical correlation between LNY and lymph node positivity (LNP) with other patient, surgical and histopathological features.

Methodology

This is a retrospective, non-interventional review of consecutive patients who underwent left-sided colonic and rectal cancer resections over a three-year period between 01 April 2018 and 31 March 2021. Descriptive and inferential statistical analyses were used. Chi-squared / Fisher exact test was used on a categorical scale between two or more groups and non-parametric setting for qualitative data analysis.

Results

A total of 102 patients were included in the study. No statistical correlation was found between the age of the patient with the LNY, LNP, location of the tumor, type, and urgency of the operation. LNY ranged between one and 43 nodes (median (interquartile range (IQR)) 17, 8). There was no statistically significant difference in LNY between laparoscopic surgery (LS) and open surgery (OS) (p=0.1449). Significant statistical correlation was identified between LNP and completeness of resection (CoR) (p=0.039), vascular invasion (VI) (p<0.001), perineural invasion (PI) (p<0.001), and circumferential resectional margin involvement (CRMI) (p=0.039).

Discussion

LNY and LNP are important prognostic indices in colorectal cancer. Patient age, tumor location, the urgency of surgery, and consultant experience did not significantly impact the LNY. Our study showed a positive correlation between LNP and CRMI, VI and PI comparable to literature. Contrary to other studies, we found no statistical significance between LS vs. OS and LNY. Whether 12 nodes per patient is an appropriate level remains controversial.

## Introduction

Colorectal cancer (CRC) is a significant cause of cancer‐related deaths worldwide [1] and is the third most common cause of cancer deaths in the United Kingdom (UK) [2,3]. Men are more likely to have a higher incidence of CRC in older age groups. However, there is only a small difference in the incidence rates between men and women aged less than 40 years [4,5]. Data from the UK suggests that men have a higher proportion of rectal cancers and women right-sided colon cancers [5].

The status of lymph node metastasis is a key factor in predicting the patient's CRC prognosis. The American Joint Commission on Cancer (AJCC) recommends that at least 12 lymph nodes be examined for each surgical specimen of CRC [6]. The lymph node ratio (LNR) is also a prognostic factor that is inversely correlated with survival outcomes [7-9]. Although the average number of lymph nodes evaluated has risen in the past decades through better surgical standards, the rate of node-positive colon cancer, surprisingly, has not increased [10,11].

Aims and objectives

We aimed to analyze the demographics of left-sided colonic and rectal cancers in a single institution. We studied the correlation between patient age with lymph node yield (LNY), lymph node positivity (LNP), location of the tumor, type, and urgency of the operation. We looked to identify any statistical correlation between the location of the tumor with the urgency, type of operation, LNY, and LNP. We aimed to identify any significant difference in LNY between laparoscopic surgery (LS) and open surgery (OS). We evaluated the correlation between LNY and LNP with tumor (T) stage, completeness of resection (CoR), vascular invasion (VI), perineural invasion (PI), degree of differentiation (DoD), consultant experience (CE), and CRM involvement (CRMI).

## Materials and methods

This is a retrospective, non-interventional review of consecutive patients who underwent left-sided colon and rectal cancer resection over a three-year period (01 April 2018 and 31 March 2021) at a single institution. For this study, we defined left-sided colorectal cancers as descending, sigmoid, rectosigmoid, and rectal cancers based on pre-operative imaging. Both elective and emergency cases were included in the study. Right colon, transverse colon, and splenic flexure cancers were excluded from the study. Patients with polyp cancers and incomplete data were also excluded from the study.

Data was retrieved from the local hospital cancer database, and after applying the inclusion and exclusion criteria, all the data was formatted in a datasheet using Microsoft Excel (Microsoft® Corp., Redmond, WA). Data collection variables included demographic data, i.e., age, gender, and American Society of Anaesthesiologists (ASA). Further data was collected from the histology reports and individual patient operation notes using the hospital's electronic patient records. After initial data collection, the data was checked and verified independently by two authors to eliminate the risk of error. The study was registered locally as an audit after approval from the colorectal cancer lead. Ethics committee approval was not required as it was a retrospective non-interventional study.

Descriptive and inferential statistical analysis was carried out in the present study. Continuous variables were presented on Mean and Standard Deviation (SD) while categorical variables as Number (n) and Percentage (%). Significance was assessed at a 5% level of significance. Chi-square / Fisher Exact test was used to find the significance of study parameters on a categorical scale between two or more groups and non-parametric setting for Qualitative data analysis. Fisher exact test was used for very small cell samples. P-value (p)<0.05 was considered statistically significant. Statistical software, namely SPSS 22.0 (IBM Corp., Armonk, NY) and R environment version 3.2.2, were used for the data analysis.

## Results

A total of 102 patients were included in the study, of which 63 (61.8%) were males and 39 (38.2%) were females. The age range was 34 to 88 years (Mean + SD: 66.5 + 11.5 years) (Table 1).

**Table 1:** Patient demographics ASA: American Society of Anaesthesiologists

Variables	No. of Patients (n=102)	%
Age in Years		
Up to 50	7	6.9
51-60	25	24.5
61-70	32	31.4
71-80	25	24.5
>80	13	12.7
Gender		
Female	39	38.2
Male	63	61.8
ASA grade		
ASA 1	0	0.0
ASA2	66	64.7
ASA3	36	35.3
ASA 4	0	0.0
ASA 5	0	0.0

LNY and LNP on histology were noted for all patients. We analyzed the correlation of patient age with the LNY, LNP, tumor location, type, and urgency of the operation. There was no statistically significant correlation (Table 2).

**Table 2:** Correlation of patient age with the LNY, LNP, tumor location, type, and urgency of the operation LNY: Lymph node yield LNP: Lymph node positivity

Variables	Age in Years (n=102)					Total	P-Value
	Up to 50	51-60	61-70	71-80	>80		
Location of tumor							
Sigmoid	2(28.6%)	12(48%)	11(34.4%)	15(60%)	5(38.5%)	45(44.1%)	0.406
Recto sigmoid	1(14.3%)	5(20%)	4(12.5%)	1(4%)	1(7.7%)	12(11.8%)	
Rectum	4(57.1%)	8(32%)	17(53.1%)	9(36%)	7(53.8%)	45(44.1%)	
Urgency of surgery							
Elective	5(71.4%)	18(72%)	27(84.4%)	22(88%)	10(76.9%)	82(80.4%)	0.543
Emergency	2(28.6%)	7(28%)	5(15.6%)	3(12%)	3(23.1%)	20(19.6%)	
Type of surgery							
Laparoscopic	6(85.7%)	23(92%)	29(90.6%)	19(76%)	9(69.2%)	86(84.3%)	0.336
Open	1(14.3%)	1(4%)	3(9.4%)	4(16%)	3(23.1%)	12(11.8%)	
Converted	0(0%)	1(4%)	0(0%)	2(8%)	1(7.7%)	4(3.9%)	
LNY							
1-10	0(0%)	3(12%)	6(18.8%)	2(8%)	3(23.1%)	14(13.7%)	0.121
11-20	3(42.9%)	10(40%)	20(62.5%)	16(64%)	9(69.2%)	58(56.9%)	
21-30	2(28.6%)	9(36%)	5(15.6%)	6(24%)	0(0%)	22(21.6%)	
31-40	1(14.3%)	2(8%)	1(3.1%)	0(0%)	1(7.7%)	5(4.9%)	
41-50	1(14.3%)	1(4%)	0(0%)	1(4%)	0(0%)	3(2.9%)	
LNP							
0	3(42.9%)	19(76%)	21(65.6%)	14(56%)	8(61.5%)	65(63.7%)	0.603
1-2	2(28.6%)	3(12%)	6(18.8%)	6(24%)	5(38.5%)	22(21.6%)	
3-5	2(28.6%)	2(8%)	3(9.4%)	4(16%)	0(0%)	11(10.8%)	
>5	0(0%)	1(4%)	2(6.3%)	1(4%)	0(0%)	4(3.9%)	
Total	7(100%)	25(100%)	32(100%)	25(100%)	13(100%)	102(100%)	

There was no significant statistical correlation between the location of the tumor with the urgency (p=0.81), type of operation (p=0.86), LNY (p=0.48), and LNP (p=0.92). We found no statistical correlation between the urgency of surgery with LNY (p=0.25) and LNP (p=0.91).

LNY ranged between 1 and 43 nodes with a median (IQR) of 17 (8) in this study. We analyzed the difference in LNY between laparoscopic surgery (LS) and open surgery (OS). LNY in LS ranged between 1 and 43 nodes (median (IQR): 17(8)). For OS, LNY ranged between 8 and 27 nodes (median (IQR): 13.5(5.5)). There was no statistically significant difference (P=0.1449, Mann-Whitney test).

We found no statistical correlation between T stage and LNY (p=0.08) and LNP (p=0.12). CoR was split into R0, R1, and R2 and studied against LNY and LNP (Table 3). There were no R2 resections in our study. There was no statistical significance between CoR and LNY (p=1). However, a direct correlation was found between the CoR and LNP (p=0.039). Vascular invasion (VI) was correlated with the LNY and LNP (Table 4). Although there was no statistical significance between VI and LNY (p=0.22), there was a statistical significance between the LNP and VI (p<0.001). Perineural invasion (PI) was correlated with the LNP and LNY (Table 5). Although there was no statistical significance between PI and LNY (p=0.53), there was a statistical significance between LNP and PI (p<0.001).

**Table 3:** Correlation between LNY and LNP with CoR ** Statistically significant LNY: Lymph node yield LNP: Lymph node positivity

Variables	Completeness of Resection (CoR)		Total	P-Value
	R0	R1		
LNY				
1-10	14(13.9%)	0(0%)	14(13.7%)	1.000
11-20	57(56.4%)	1(100%)	58(56.9%)	
21-30	22(21.8%)	0(0%)	22(21.6%)	
31-40	5(5%)	0(0%)	5(4.9%)	
41-50	3(3%)	0(0%)	3(2.9%)	
LNP				
0	65(64.4%)	0(0%)	65(63.7%)	0.039**
1-2	22(21.8%)	0(0%)	22(21.6%)	
3-5	11(10.9%)	0(0%)	11(10.8%)	
>5	3(3%)	1(100%)	4(3.9%)	
Total	101(100%)	1(100%)	102(100%)	

**Table 4:** Correlation between LNY and LNP with VI ** Statistically significant LNY: Lymph node yield LNP: Lymph node positivity

Variables	Vascular invasion (VI)		Total	P-Value
	No	Yes		
LNY				
1-10	13(17.1%)	1(3.8%)	14(13.7%)	0.219
11-20	43(56.6%)	15(57.7%)	58(56.9%)	
21-30	14(18.4%)	8(30.8%)	22(21.6%)	
31-40	3(3.9%)	2(7.7%)	5(4.9%)	
41-50	3(3.9%)	0(0%)	3(2.9%)	
LNP				
0	56(73.7%)	9(34.6%)	65(63.7%)	<0.001**
1-2	14(18.4%)	8(30.8%)	22(21.6%)	
3-5	5(6.6%)	6(23.1%)	11(10.8%)	
>5	1(1.3%)	3(11.5%)	4(3.9%)	
Total	76(100%)	26(100%)	102(100%)	

**Table 5:** Correlation between LNY and LNP with PI ** Statistically significant LNY: Lymph node yield LNP: Lymph node positivity

Variables	Perineural Invasion (PI)		Total	P-Value
	No	Yes		
LNY				
1-10	14(14.7%)	0(0%)	14(13.7%)	0.525
11-20	53(55.8%)	5(71.4%)	58(56.9%)	
21-30	21(22.1%)	1(14.3%)	22(21.6%)	
31-40	4(4.2%)	1(14.3%)	5(4.9%)	
41-50	3(3.2%)	0(0%)	3(2.9%)	
LNP				
0	65(68.4%)	0(0%)	65(63.7%)	<0.001**
1-2	19(20%)	3(42.9%)	22(21.6%)	
3-5	9(9.5%)	2(28.6%)	11(10.8%)	
>5	2(2.1%)	2(28.6%)	4(3.9%)	
Total	95(100%)	7(100%)	102(100%)	

The correlation between LNY and LNP and circumferential resection margin involvement (CRMI) was analyzed (Table 6). There was no significant correlation between stapler doughnuts and degree of differentiation with LNY (p=1) and LNP (p=0.60). There was no significant correlation between the degree of differentiation with LNY (p=0.28) and LNP (p=0.29). There was no statistically significant correlation between the LNY and CRMI. However, the correlation between CRMI and LNP was statistically significant (p=0.039).

**Table 6:** Correlation between LNY and LNP with CRMI ** Statistically significant CRMI: Circumferential resection margin involvement LNY: Lymph node yield LNP: Lymph node positivity

Variables	CRM Involvement (CRMI)		Total	
	No	Yes		
LNY				
1-10	14(13.9%)	0(0%)	14(13.7%)	1.000
11-20	57(56.4%)	1(100%)	58(56.9%)	
21-30	22(21.8%)	0(0%)	22(21.6%)	
31-40	5(5%)	0(0%)	5(4.9%)	
41-50	3(3%)	0(0%)	3(2.9%)	
LNP				
0	65(64.4%)	0(0%)	65(63.7%)	0.039**
1-2	22(21.8%)	0(0%)	22(21.6%)	
3-5	11(10.9%)	0(0%)	11(10.8%)	
>5	3(3%)	1(100%)	4(3.9%)	
Total	101(100%)	1(100%)	102(100%)	

The operating surgeon's consultant experience (CE) was classified as less than 10 years and 10 or more years. There was no statistical significance between consultant experience and LNY (Table 7).

**Table 7:** Correlation between LNY and LNP with CE LNY: Lymph node yield LNP: Lymph node positivity

Variables	Consultant Experience (CE)		Total	P-Value
	<10 Years	>=10 Years		
LNY				
1-10	10(14.3%)	4(12.5%)	14(13.7%)	0.568
11-20	36(51.4%)	22(68.8%)	58(56.9%)	
21-30	17(24.3%)	5(15.6%)	22(21.6%)	
31-40	4(5.7%)	1(3.1%)	5(4.9%)	
41-50	3(4.3%)	0(0%)	3(2.9%)	
Total	70(100%)	32(100%)	102(100%)	

## Discussion

This study represents the demographic and oncological outcomes for descending, sigmoid, rectosigmoid, and rectal cancers at a single institution in the east of England. Critical prognostic factors after colorectal cancer resection are the LNY and LNP. Not only are LNY and LNP important for accurate staging and quality of surgical intervention, but they also have prognostic value in guiding the further management of the patients [12, 13]. A retrospective study [14] showed a statistically significant increase in LNY between 1997 and 2013. The cause is multifactorial, with the main one being increased awareness among surgeons about the significance of LNY. No difference was found in LNY between LS and OS. However, Puckett et al. [15] study showed that an open operative approach, when compared to the laparoscopic approach, was associated with 15% greater odds of retrieval of >12 LNs (OR 1.148; 95% CI (1.035-1.272); p = 0.008).

Our study showed a positive correlation between LNP and CRMI, VI, and PI. The results are comparable to a study by Balbaa et al. [16], which showed that a statistically significant correlation exists between CRMI and LNP (p=0.001), and VI (p=0.021) and PNI (p=0.04). There was no significant association between the T stage and LNY and LNP in our study. However, some previous studies have shown a direct correlation between the T stage and LNY [17-20]. Young age, reported to be associated with LNY [21,22], was not found in our study. In 2011, Rivard and Hochman [23] claimed no statistically significant difference between the LNY during laparoscopic versus open surgery (17.4 vs. 18.5; P=0.5920). Widmar et al. [24] hypothesized that robotic surgery would lead to higher LNY when compared to LS and OS with its increased dexterity and visualization. However, this study showed no statistical significance between LS and OS with LNY and LNP. We point out that our study did not include robotic procedures.

Dutch registry analysis with over 17000 patients studied suggested that LNY ≥ 12 is significantly associated with age, pT stage, tumor subsite, and priority of surgery. They hypothesize that a significant increase in the LNY over the period of the study was observed, probably reflecting the effect of national programs initiated by the Danish Colorectal Cancer Group [25]. Our study is limited to a single institution and found no significance between LNY with age, the urgency of operation, type of operation, or tumor subsite. Rivard and Hochman [23] found that the number of lymph nodes harvested decreased with increasing ASA grade and neoadjuvant radiotherapy. Our study did not investigate that aspect.

The number of lymph nodes recovered from resection specimens is dependent on several factors, such as surgical technique, tumor location, and length of bowel resected [26]. It is mainly dependent on the diligence and skill of the pathologist [27]. There is sizeable variation between different pathology laboratories about the number of lymph nodes examined histologically, as found in a Dutch nationwide study [28]. Whether 12 nodes per patient is an appropriate level is controversial. In this study, the LNY ranged between 1 and 43 nodes with a median (IQR): 17(8). It did not correlate with the type of surgery or consultant experience. It may be appropriate that guidance be revised to a higher number, leading to a higher LNP [29]. Evans et al. [30] support the argument that with a higher proportion of cases classified as LNP, cases rise to 36 nodes/patient. These findings need to be looked at with caution because higher LNY observed may be a consequence of the severe disease, rather than patients with lower LN yields having missed nodal metastases [30].

## Conclusions

LNY and LNP are important prognostic indexes in colorectal cancer. Patient age, tumor location, the urgency of surgery, and consultant experience did not significantly impact the LNY in this study. Our study showed a positive correlation between LNP and CRMI, VI, and PI comparable to previously published literature. This is useful to have in perioperative planning and discussion. A few studies published suggest that LNY in laparoscopic surgery is superior to open surgery. Contrary to these studies, we found no statistical significance between LS vs. OS and LNY. A meticulous operation following the embryonic planes of dissection irrespective of the access will yield better results as our study suggests. Whether 12 nodes per patient is an appropriate level is controversial. LNY depends on several factors such as severity of disease, neoadjuvant therapy, pathologist's skill and tumor location. Attributing LNY to surgical technique or access alone would be an oversimplification.
